# Supplementary material for: Rationally Improving Doramectin Production in Industrial Streptomyces avermitilis Strains
Source: Bioengineering (Basel). 2023 Jun 20;10(6):739. doi: 10.3390/bioengineering10060739 (PMC10295569; doi:10.3390/bioengineering10060739)
Supplement: Supplementary file 1 [file bioengineering-10-00739-s001.zip › bioengineering-2317341-supplementary.pdf]

Supplementary materials

Rationally improving doramectin production in industrial *Streptomyces avermitilis* strains

Fujun Dang[1], Qingyu Xu[1], Zhongjun Qin[1] and Haiyang Xia[1,2,*]

[1] Key Laboratory of Synthetic Biology, the Center of Excellent Plant Molecular Sciences, the Chinese Academy of Sciences, Shanghai, 200032

[2] Institute of Biopharmaceuticals, Taizhou University, Taizhou, 317000, China

* Correspondence: hyxia@tzc.edu.cn.

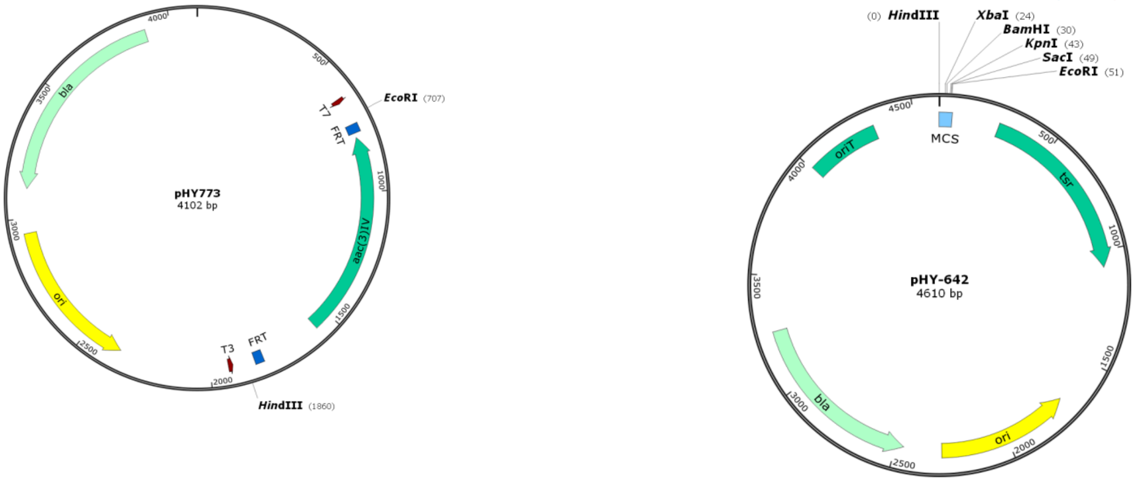

(A) (B)

Figure S1. Physical maps of plasmids pHY773 and pHY-642. (A), pHY773 containing the FRT-*aac(3)IV*-FRT cassette. (B), pHY-642 can be used as a shuttle vector for gene deletion in *Streptomyces*.

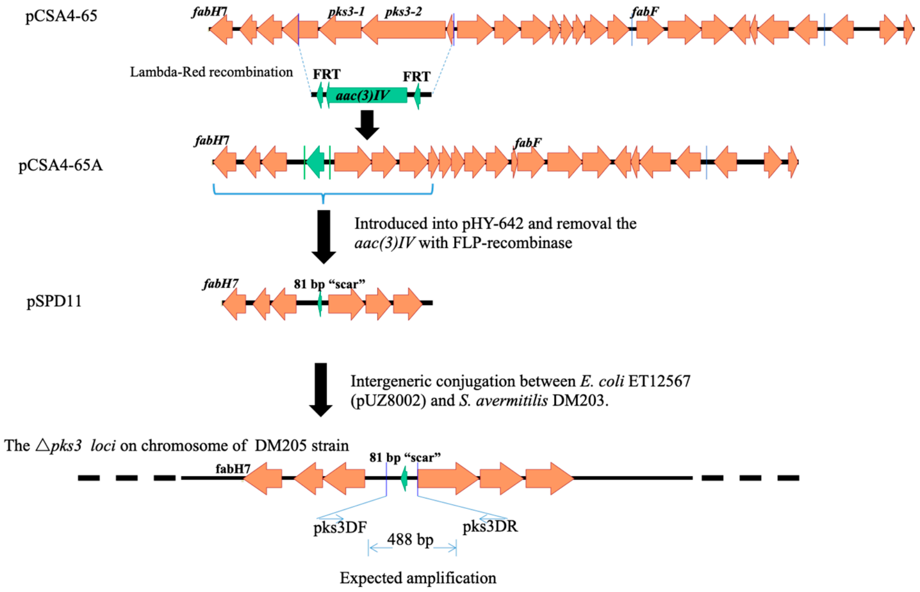

(A)

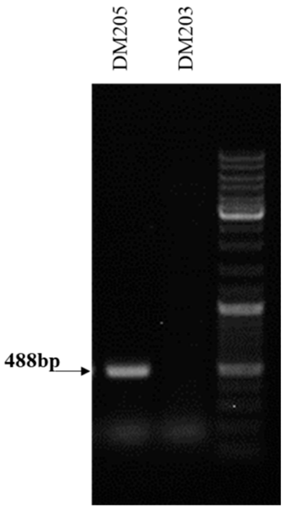

(B)

Figure S2. Markerless deletion of the *pks3* cluster from *S. avermitilis* DM203. (A), Schematic strategy for markerless deletion of the *pks3* cluster from strain DM203. Plasmid pCSA4-65A resulted from replacement of the *pks3* cluster with the FRT-*aac(3)*IV-FRT cassette on cosmid pCSA4-65 by lambda Red recombination. The fragment contains the FRT-*aac(3)IV*-FRT cassette introduced into pHY-642. The *aac(3)IV* on the resulting plasmid was cleaved by FLP-recombinase to generate pSPD11. Plasmid pSPD11 was introduced into strain DM203 by intergeneric conjugation. The double-crossover strains were selected by restreaking conjugants to antibiotic-free ISP2 medium. Clones with loss of thiostrepton resistance were verified by PCR amplification with the pks3DF and pks3DR primer pair. (B), PCR verification of *pks3* deletion in DM205 with the pks3DF and pks3DR primer pair.

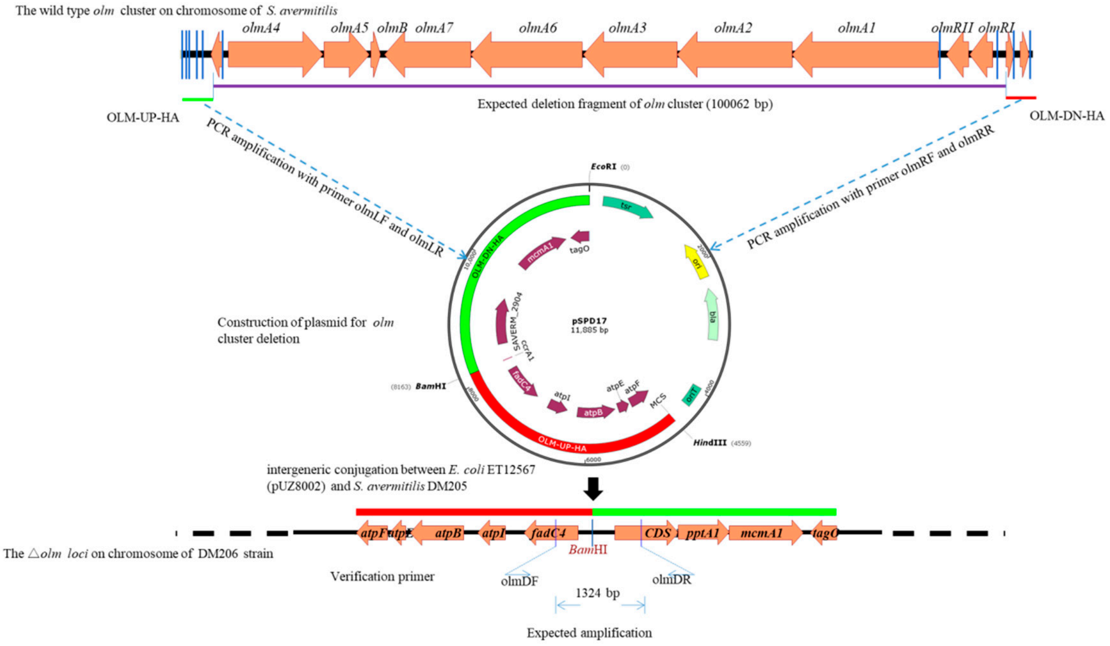

(A)

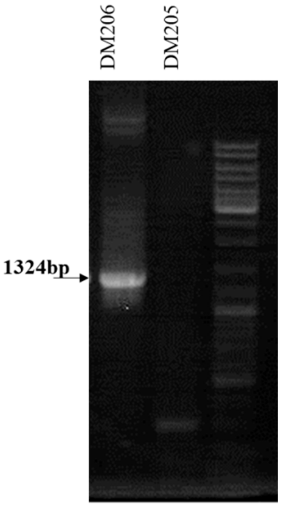

(B)

Figure S3. Schematic strategy for markerless deletion of the *olm* cluster from *S. avermitilis* DM205. (A), Diagram of the construction of pSPD17 for deleting the *olm* cluster. The upstream and downstream homologous arms were amplified and introduced into pHY-642 to generate pSPD17. Plasmid pSPD17 was introduced into strain DM205 by intergeneric conjugation. The double-crossover strains were selected by restreaking conjugants to antibiotic-free ISP2 medium. Clones with loss of thiostrepton resistance were verified by PCR amplification with the olmDF and olmDR primer pair. (B), PCR verification of *olm* deletion in DM206 with the olmDF and olmDR primer pair.

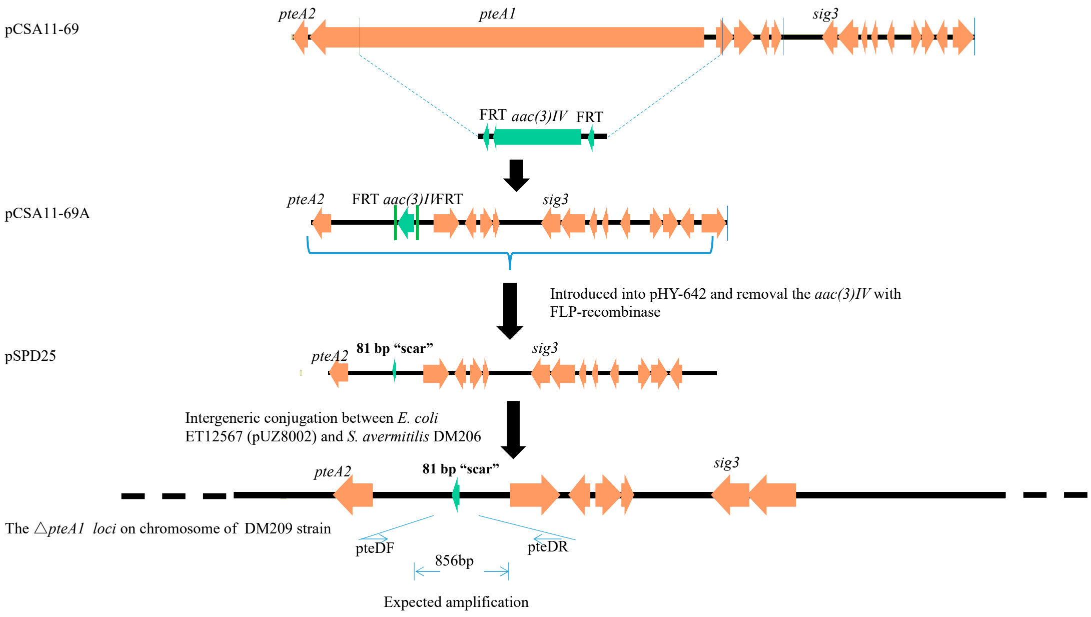

(A)

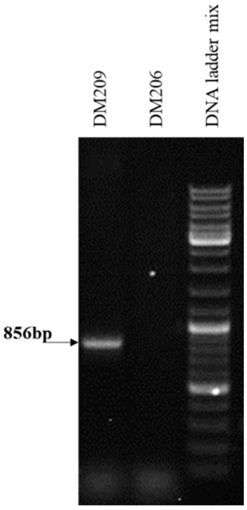

(B)

Figure S4. Markerless deletion of a partial *pte* cluster from *S. avermitilis* DM206. (A), Schematic strategy for markerless deletion of the *pte* cluster from strain DM206. Plasmid pCSA11-69A resulted from the replacement of a partial *pte* cluster with the FRT-*aac(3)IV*-FRT cassette on cosmid pCSA4-65 by lambda Red recombination. The fragment contains the FRT-*aac(3)IV*-FRT cassette introduced into pHY-642. The *aac(3)IV* cassette on the resulting plasmid was cleaved by Flp-recombinase to generate pSPD25. Plasmid pSPD25 was introduced into strain DM206 by intergeneric conjugation. The double-crossover strains were selected by restreaking conjugants to antibiotic-free ISP2 medium. Clones with loss of thiostrepton resistance was verified by PCR amplification with the pteDF and pteDR primer pair. (B), PCR verification of *pte* deletion in DM209 with the pteDF and pteDR primer pair.

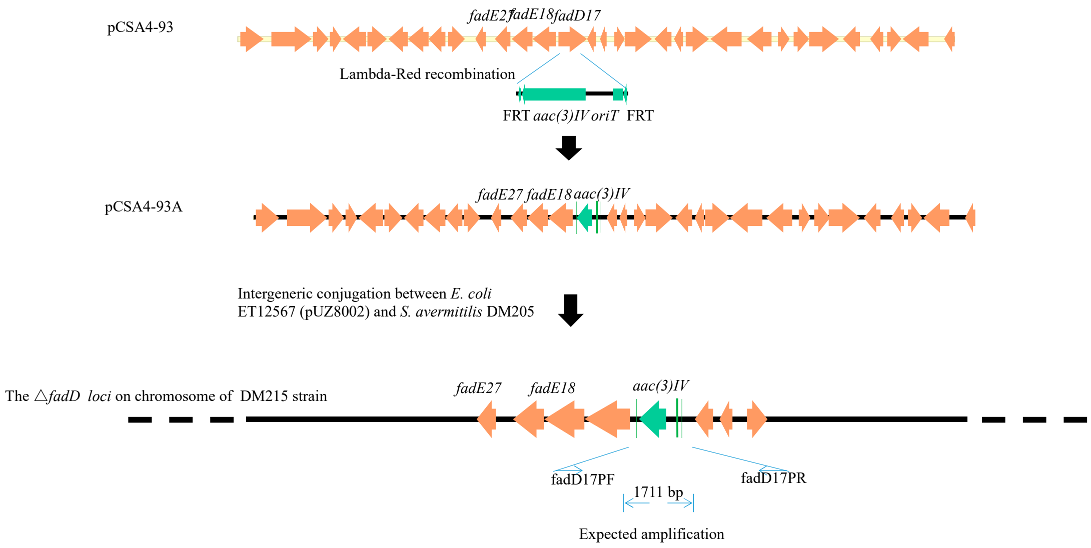

(A)

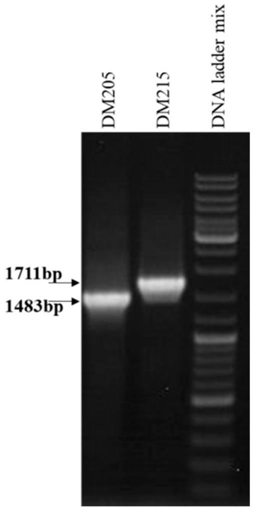

(B)

Figure S5. Disruption of *fadD17* from *S. avermitilis* DM205. (A), PCR-targeting *fadD* from strain DM205. Plasmid pCSA4-93A resulted from the replacement of *fadD17* with the FRT-*aac(3)IV*-*oriT*-FRT cassette on cosmid pCSA4-65 by lambda Red recombination. Plasmid pCSA4-93A was introduced into strain DM205 by intergeneric conjugation. The double-crossover strains were screened out by selection of apramycin-resistant and kanamycin-sensitive conjugants. The positive clones were verified by PCR amplification with the fadD17PF and fadD17PR primer pair. (B), PCR verification of *fadD17* deletion in DM215 with the fadD17PF and fadD17PR primer pair.

Table S1. Putative CoA ligase-encoding genes in *Streptomyces avermitilis.*

ID	Start	End	Definition
SAV_377	445913	447400	putative acyl-CoA synthetase, long-chain fatty acid:CoA ligase
SAV_605	760552	758996	putative acyl-CoA synthetase, long-chain fatty acid:CoA ligase
SAV_1104	1388246	1390171	putative acyl-CoA synthetase, long-chain fatty acid:CoA ligase
SAV_1246	1544829	1546583	putative acyl-CoA synthetase, long-chain fatty acid:CoA ligase
SAV_1257	1559656	1560951	putative phenylacetate:CoA ligase
SAV_1258	1562499	1561000	putative acyl-CoA synthetase, long-chain fatty acid:CoA ligase
SAV_1259	1564346	1562796	putative acyl-CoA synthetase, long-chain fatty acid:CoA ligase
SAV_1603	1970811	1969300	putative acyl-CoA synthetase, long-chain fatty acid:CoA ligase
SAV_1627	1996963	1995767	putative 2-amino-3-oxobutyrate:CoA ligase
SAV_1848	2264965	2266791	putative acyl-CoA synthetase, long-chain fatty acid:CoA ligase
SAV_3330	4145296	4144421	putative biotin apoprotein ligase
SAV_3416	4238429	4237848	putative secreted protein
SAV_3674	4548308	4548931	putative ligase
SAV_3806	4710123	4708540	putative acyl-CoA synthetase, fatty acid:CoA ligase
**FadD (SAV_3841)**	**4745600**	**4747099**	**putative cyclohex-1-ene-1-carboxylate:CoA ligase**
SAV_3864	4773500	4771926	putative 4-coumarate:CoA ligase
SAV_4206	5162486	5160810	putative acyl-CoA synthetase, long-chain fatty acid:CoA ligase
SAV_4818	5858175	5860091	putative acyl-CoA synthetase, long-chain fatty acid:CoA ligase
SAV_5225	6333369	6335009	putative acyl-CoA synthetase, long-chain fatty acid:CoA ligase
SAV_5562	6727208	6729118	putative acyl-CoA synthetase, long-chain fatty acid:CoA ligase
SAV_5723	6924728	6923259	putative acyl-CoA synthetase, long-chain fatty acid:CoA ligase
SAV_6069	7307315	7305519	putative acyl-CoA synthetase, long-chain fatty acid:CoA ligase
SAV_6612	7911728	7913395	putative acyl-CoA synthetase, long-chain fatty acid:CoA ligase
